# Pilot Data for a New Headphone-Based Assessment of Absolute Localization in the Assessment of Auditory Processing Disorder (APD)

**DOI:** 10.3390/audiolres15010012

**Published:** 2025-01-27

**Authors:** Jack Hargreaves, Julia Sarant, Bryn Douglas, Harvey Dillon

**Affiliations:** 1 Department of Audiology and Speech Pathology, The University of Melbourne, Melbourne, VIC 3010, Australia; 2 Department of Linguistics, Macquarie University, Sydney, NSW 2113, Australia

**Keywords:** absolute localization, auditory processing disorder (APD), head-related transfer function, front–back error

## Abstract

**Background/Objectives**: Localization deficit is often said to be a symptom of Auditory Processing Disorder (APD). However, no clinically viable assessment of localization ability has been developed to date. The current study presents pilot data for a new assessment of absolute auditory localization using headphones. **Methods**: Speech phrases encoded with non-individualized head-related transfer functions (HRTF) using real-time digital processing were presented to two cohorts of participants with normal hearing. Variations in the simulated environment (anechoic and reverberant) and signal to noise ratio (SNR) were made to assess each of these factors’ influences on localization performance. Experiment 1 assessed 30 young adults aged 21–33 years old and Experiment 2 assessed 28 young adults aged 21–29 years old. All participants had hearing thresholds better than 20 dB HL. **Results**: Participants performed the localization task with a moderate degree of accuracy (Experiment 1: Mean RMS error = 25.9°; Experiment 2: Mean RMS error 27.2°). Front–back errors (FBEs) were evident, contributing to an average RMS error that was notably elevated when compared to similar free-field tasks. There was no statistically significant influence from the simulated environment or SNR on performance. **Conclusions**: An exploration of test viability in the pediatric and APD-positive populations is warranted alongside further correction for FBEs; however, the potential for future clinical implementation of this measure of absolute auditory localization is encouraging.

## 1. Introduction

Within a complex auditory scene comprising multiple speakers, ambient noise, and reverberation, normal hearing adults can pinpoint sound sources of interest using auditory information alone [1]. The ability to make sense of complex auditory scenes, often referred to as spatial hearing, is largely due to a combination of the processes of auditory localization, the ability to locate sound sources [2] and spatial stream segregation (SSS), and the ability to derive distinctive auditory streams from a complex wavefront [1]. Both processes are mediated by the Central Auditory Nervous System, which can discriminate minute variations in signals received in each ear independently [2,3]. Auditory localization in particular utilizes binaural and monaural cues to define the depth, vertical angle, and horizontal angle of a sound source [2]. Prior studies have demonstrated that normal-hearing listeners have an exceptional localization ability, as reflected in broadband, free-field, frontal hemisphere absolute localization tasks, displaying average root mean square (RMS) errors of between 6 and 10 degrees [4].

The perception of sound source location relies on binaural disparities such as Interaural Timing Differences (ITD; differences in arrival time between the leading and lagging ears) and Interaural Intensity Differences (IID; differences in the intensity of sounds between the leading and lagging ears). The ITDs and IIDs are first encoded in the auditory periphery [5]. These comparative signals are then processed in separate brainstem nuclei, with ITDs in the medial superior olive and IIDs in the lateral superior olive [6,7], and are thought to converge as early as the inferior colliculus [5]. Due to head, torso, and pinna interactions, binaural cues are notably unequal across the frequency range, with IIDs being more dominant in high frequencies (>2000 Hz), as the longer wavelength of low-frequency sounds is less impacted by the head shadow effect [2,8]. Conversely, ITDs are primarily extracted from low-frequency regions (<1500 Hz), as the shorter period of high-frequency tones relative to the ITDs (from 0 to 800 μs) results in ambiguous phase differences between waveforms transduced in each ear [2,8]. Given the positioning of the ears on the head in the same horizontal plane, binaural cues predominantly help to localize a sound source in the horizontal plane [8]. However, frequency-specific attenuation and boosting created by the direction-dependent interactions of an incoming signal with the pinna generate unique spectral envelopes [9]. These monaural cues, active above 3 kHz, contribute to azimuthal localization and are most important for localization along the medial vertical plane where binaural disparities are minimized [3]. To determine auditory location, significant neural processing of the incoming signal must occur [10], primarily within the subcortical areas [1]. However, this complex computational comparative processing is less well understood, and multiple proposed neural models exist [11,12].

Some individuals experience listening difficulties in the presence of otherwise normal audiograms [13], which are notably distinct from Auditory Neuropathy, where individuals present with abnormal auditory brainstem response patterns. That is, despite normal pure tone thresholds, individuals with Auditory Processing Disorder (APD) may have difficulty in understanding speech in background noise, localizing sound sources, detecting subtle changes in prosody, and other functions [14]. APD is estimated to be present in 2–5% of the child population and 23–76% of the older adult population [13,15]; however, these estimates may be inaccurate due to a lack of diagnostic consensus.

Various audiological guidelines outline potential behavioral manifestations associated with APD populations, and all are inclusive of localization deficit [14,16,17]. The first direct investigation of this deficit [18] included 17 APD-positive children, diagnosed via the Multiple Auditory Processing Assessment (MAPA) [19] (now refined and known as the MAPA-2 [20]). A localization test was performed under headphones, simulating nine perceived source angles and assessing ITD and IIDs separately. Compared to 20 typically developing (TD) children, the performance of children with APD was significantly poorer. ITD errors were not only more common than IID errors in all APD participants, but were also higher within the APD group. Lotfi et al. [21] assessed improvement after training in 60 participants diagnosed with APD via the MAPA, finding also that APD children made more localization errors under headphones than their TD peers. A more recent study [22] used a free-field task of absolute localization with 46 APD-diagnosed children. While the majority of APD children performed normally compared to TD peers, they performed more poorly than controls at or around the midline (0 degrees of azimuth).

In a clinical setting, APD deficits are typically addressed in a modality-specific approach, with several clinical assessments being employed to isolate specific perceptual and cognitive deficits [14,16]. Perceptual assessments are designed to address the neural/bottom-up domains of auditory processing. For example, the Frequency Pattern Test (FPT), which examines the ability to identify and remember the order of tones of two different pitches, is used to assess temporal sequencing [23]. Alternately, cognitive assessments are used to assess higher order/top-down functions (e.g., the TONI-4 testis used to assess Non-verbal IQ [24]). Current AAA diagnostic criteria specify that individuals who score two standard deviations below the normative mean in at least one ear on two of the behavioral tasks have APD [14].

Despite the apparently established view that impaired localization is symptom of some types of APDs, there is currently no clinically standardized assessment of localization ability. Several types of assessment of sound source localization ability exist, collectively referred to as spatial audiometry [25], and involve the presentation of test stimuli from different locations (physical free-field or virtual) to determine the patient’s ability to process binaural information and perceive location from auditory information [26]. Localization tasks are either relative acuity tasks, used to establish the smallest minimum audible angle (MMA) between which an individual can distinguish two sound sources, or absolute accuracy tasks, used to establish absolute localization ability [27]. Free-field assessments incorporate static multi-speaker arrays with participants required to report the direction of the stimuli presentation [28], whereas virtual assessments, typically performed using headphones, use a simulated spatial environment to present stimuli [29]. Although free-field assessments are useful, the installment of a multi-speaker array is rarely viable in clinical settings. Further limitations, such as calibration issues, the repeatability of stimuli presentation, and difficulties in limiting dynamic localization cues, suggest the need for more accessible and controlled assessment methods [28]. Spatial simulation technology, actively used in research and virtual gaming, is an easy-to-use alternative that enables better control of testing conditions in a clinical setting.

As there are currently no tools available to address the need for accurate identification of disabilities in absolute localization, the current study proposes a novel headphone-based assessment of absolute localization using virtual auditory environment technology. The aim of this study was to provide pilot data for typically developed young adults using a novel localization task (Measurement of Azimuthal Perception (MAP)) to assist with future assessments of binaural interaction ability to identify individuals in need of APD interventions.

## 2. Experiment 1

### 2.1. Materials and Methods

#### 2.1.1. Participants

Participants were 30 young adults (70% female; mean [range] age 25 [21–33] years) recruited from the Macquarie University Masters of Clinical Audiology 2020 intake. With this sample size, mean performance under each condition could be estimated with a standard error of the mean equal to 18% of the standard deviation of the spread of performance in the group, a level of precision considered adequate. A Latin Square design was used to allocate the test conditions, with equal numbers of participants assessed under each combination of test conditions.

Participants were required to have (1) self-reported bilateral hearing status within normal limits, (2) no previously reported diagnosis of a learning, attention, or auditory processing disorder, (3) adequate proficiency in English to understand the speech-based stimulus, and (4) competent computer-based literacy. COVID-19 pandemic restrictions prevented in-person assessments, so only individuals who had passed a full audiometric examination within the previous 12 months as part of their audiology degree were eligible for participation. Written informed consent was obtained from all participants after approaching them via email.

#### 2.1.2. Stimuli Materials

A series of 30 pre-recorded short speech phrases (mean [range] duration 1.19 [0.74–1.86] s; Appendix A) recorded by a native English-speaking male were used in the MAP protocol. Short speech phrases were selected to ensure access to both IID and IID cues for horizontal localization. Stimuli were recorded under semi-anechoic conditions at a sample rate of 44.2 kHz using an AKG C214 large diaphragm condenser microphone. Stimuli were recorded using a close-microphone technique and Steinberg UR22C USB audio interface with 24-bit resolution using a Dell laptop (model XPS). Stimuli were normalized to a consistent -23 re dBFS level during recording to ensure full spectral dynamics were captured. Simulated source angle was encoded to the anechoic recordings by convolving each recording with a non-individualized head-related impulse response (HRIR), commonly used to encode localization cues in simulated auditory environments, that corresponded to a desired horizontal azimuth. A full set of binaural HRIRs corresponding to each 1-degree azimuth of a 360-degree array were obtained from a free online database in MAT format [30], recorded at a distance of 2 m in a sound-treated anechoic chamber using a KEMAR GRAS 45BB head and torso mannequin.

All free-field HRIR spectral recordings also required additional cancelation of the ear canal resonance that was present under the headphones. To achieve this, an impulse response was recorded from a KEMAR GRAS 45BB head and torso mannequin using Sennheiser HD215 headphones. The inverse transfer function corresponding to this impulse response was convolved with the binaural HRIRs at each azimuth. The same headphone correction was applied to both the left and right channels of the binaural HRIRs. The resulting headphone-corrected binaural HRIRs were then convolved with the monaural speech recordings using the Matlab *fftfilt* function.

#### 2.1.3. Instrumentation

Stimulus/paradigm presentation was conducted over the internet due to COVID-19 pandemic restrictions. Zoom Video Communications software (V 5.5.5) was used to communicate with each participant remotely, streaming using the stereo high-fidelity audio share-type, an option that prioritizes minimal distortion and a high degree of likeness to the original audio source. This share-type constitutes a conversion-free direct stereo audio stream from the host’s computer soundcard to the participant’s computer soundcard at an audio output frequency of >36 kHz. The average packet loss, the loss of data between the origin and source of streaming, was tracked in real-time and equated to 0% average loss for the duration of each assessment administration. Camera stream capability was disabled for both parties; therefore, any shared outgoing packets were limited to transmission of the presenter screen and direct high-fidelity stereo audio. Incoming packets were limited to each participant’s verbal response streamed via microphone audio alone. Each participant was permitted to use the transducer with which they felt most comfortable. Prior to remote testing, all participants were required to seek a quiet distraction-free environment.

A 2015 MacBook Pro (2.9 GHz Intel Core i5 computer processing unit, 16 GB memory, macOS Mojave, version 10.14.6) was used for audio-visual presentation. MATLAB software (version R2020b) [31] was used to present both MATLAB-based applications and graphic display interfaces (see MCL and MAP test paradigms). Participants were asked to adjust their computer volume to 75% capacity before any testing. This was implemented as a gain staging procedure to reduce any potential for digital distortion and/or unwanted signal noise.

#### 2.1.4. Condition/Environment

In addition to processing sounds as if they were presented in an anechoic environment, they were processed to simulate reverberation by combining the direct wave from the target direction with 500 reflections of it, arriving from random directions and random delays. Each individual reflection was simulated to arrive from any random direction within a 360-degree azimuthal field of 1-degree increments. The first reflection arrived 10 milliseconds after the direct sound of the original stimulus, with remaining reflections arriving between 10 and 200 milliseconds. As in real rooms, the additional reflections accompanying each stimulus increased in density (reflections/ms) as the square of the delay time. Each reflection was implemented as the HRIR appropriate to its randomly selected direction, attenuated by an amount appropriate to its pseudo-randomly selected delay. The attenuation was calculated from an exponential decay that reached 60 dB of attenuation 200 after the direct sound arrived (i.e., a room reverberation time of 0.2 s). This direct sound and the sum of the reverberant components of the HRIR were weighted to have equal power, therefore simulating the listener being positioned at the critical distance from the source and summed to produce a composite HRIR. This composite HRIR was convolved with the original speech stimulus waveform.

Background noise simulation was achieved through the addition of speech-like babble using randomly combined consonant–vowel syllables spoken by a native English-speaking female. Recordings were of 10 consonants preceding each of three vowels, giving 30 different consonant–vowel syllables with a typical duration of 400 ms. In each trial, five randomly chosen syllables were concatenated, with a 300 ms gap between syllables to produce a five-syllable pseudoword of typical duration 3.2 s. Five such pseudowords were created, and each pseudoword was convolved with an HRIR appropriate to −90°, −45°, 0°, 45°, or 90°. Thus, one pseudoword appeared to come from each of these five directions. Each pseudoword was preceded by a silent interval randomly chosen in a range from 0 to 1.5 s, and these zero-padded stimuli were added to produce a composite masker. The composite masker commenced 2 s prior to the start of the target stimulus.

#### 2.1.5. Most Comfortable Loudness Protocol (MCL)

Given the COVID-enforced need for an internet-based research methodology, intensity consistency and transducer calibration were unable to be applied directly. Instead, the “Most Comfortable Loudness” (MCL) application was administered to each participant before the primary assessment, with the interactive graphic display controlled by the tester. The MCL comprised a series of pre-recorded vocalizations of the digits one through nine spoken by a native English-speaking male. For the duration of the MCL assessment, 5 randomly selected digits were presented to the participants, who were required to report on the audibility of the stimuli. Five possible choices were given: (1) “up a lot”, (2) “up a little”, (3) “that’s perfect”, (4) “down a little”, and (5) “down a lot”. Stimuli intensity was adjusted according to participant choice (by ±6, ±2, or 0 dB), and the spoken digit sequence was re-played, with participants encouraged to experiment with different intensity levels to establish an audible presentation level. This continued until participants reported “that’s perfect” for two consecutive presentations or until 10 trials were completed, after which the average attenuation needed to achieve MCL was calculated. This same attenuation was used to control the intensity of the target speech stimuli presentations for each participant. 

#### 2.1.6. Measurement of Azimuthal Perception (MAP)

**Figure 1:**
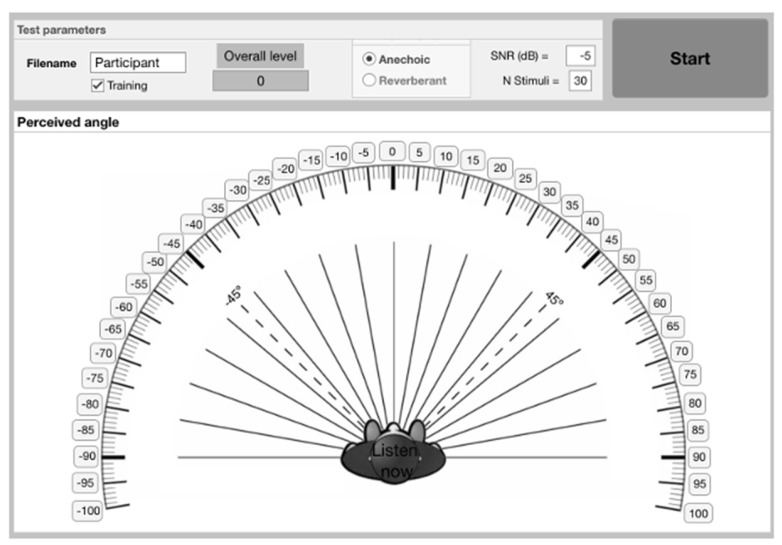
Graphical display and interactive user interface for the Measurement of Azimuth Perception (MAP) test software as generated and run using Mathworks MATLAB software. Participant perceived azimuth was confirmed by selecting 1 of the 41 available response boxes arranged in 5-degree increments of azimuth. User controlled parameters determine (1) test condition variables, (2) “overall level” consistency as defined by the MCL correction dB factor, and (3) the conditioning paradigm “training” checkbox.

The MAP protocol was designed to assess the absolute localization ability of normal-hearing young adult listeners. Prior to testing, participants were instructed to perceive all speech stimuli as arising externally from their head and asked to maintain a still front-facing head position. They were seated in front of a graphic display, as shown in Figure 1. The graphic display presents potential source angles in 5° increments covering the frontal hemisphere of azimuth, but extending from −100 to +100°, with 0 degrees representing sources directly in front of the listener.

Stimulus presentation commenced with a “training” paradigm consisting of 10 stimulus presentations per participant. As these training stimuli were presented, a simultaneous red light appeared on the graphic display, representing the source direction. This training protocol served two purposes: (1) ensuring each participant was familiar with the assessment protocol, and (2) allowing for perceptual transfer/familiarity between perceiving each simulated sound-source presentation and interpreting the corresponding numeric angle on their 2D display of azimuth.

After the training paradigm, participants were assessed in each of six test conditions, comprising three different SNRs (+5 dB, −5 dB, and speech in quiet) in an anechoic and reverberant simulated environment. A Latin square design was used to control the ordering effects of the assignment of test conditions. Each condition presented 30 stimuli, and there was a short break between conditions. A readying protocol was employed 2 s before each stimulus presentation in the form of a flashing red “listen now” on the graphic display to encourage participant attention. A verbal response was required after each stimulus source presentation, indicating the source direction perceived by the participant. The experimenter selected the corresponding directional box, which illuminated to confirm to both experimenter and participant which box was selected. All responses were saved after each condition was completed.

As shown in Figure 1, participants could choose any potentially perceived/verbal response within a range of between −100 degrees and +100 degrees in 5° azimuthal increments, corresponding to any of the available boxes graphically displayed. However, source stimuli only arose from the frontal hemisphere within a range from −90° to +85°. An additional 10 degrees of response choices were given to combat potential subjective bias imposed by the maximal ends of lateral presentation on the listener’s perceived azimuth. The 30 stimuli presentations in all conditions were categorized as arising from within six sectors: frontal A (from −30 to −5 degrees), frontal B (from 0 to +25 degrees), middle A (from −60 to −35 degrees), middle B (from +30 to +55 degrees), lateral A (from −90 to −65 degrees), and lateral B (from +60 to +85 degrees). Presentation programming randomly assigned a sector as an element before randomly assigning an element within that sector for stimulus presentation with the corresponding HRIRs imposed. Unknown to each participant, all 30 stimulus presentations within each condition were therefore spread evenly amongst each of these six sectors, resulting in five stimulus presentations per sector for each condition.

#### 2.1.7. Statistical Analysis

Statistica statistical software (Version 13.3) [32] was used for all data analyses. Cross-sectional within-subject quantitative data of three factors (SNR, environment, and sector) were analyzed. The accuracy of each participant’s response was calculated as the root mean square (RMS) value of the difference between speech-phrase-sourced azimuth and participant-perceived azimuth. Participant response data were also computed in a visual format following each condition as a graphic function of perceived azimuth over source azimuth, designated as a perception function. The corresponding sectors of A and B were conjoined for all analyses, resulting in three sectors (frontal, middle, and lateral). Use of a Latin square design saw all combinations of factors presented across all participants. Analysis of test order’s influence on RMS error (averaged across the SNR, environment, and sector) was calculated, and a correction factor was obtained and applied to further analyses. 

The mean and standard deviation of RMS error as a function of SNR and test environment were calculated. Mean and standard deviation of each individual condition’s perceptual function for all participants was calculated following test order correction factor application to form a single perceptual function corresponding to each condition. A three-way analysis of variance (ANOVA) was conducted with the SNR, test environment, and presentation sector (frontal, middle, and lateral) as repeated-measures independent variables and RMS error as the dependent variable. A Pearson correlation coefficient was computed to assess whether there was a linear relationship between each participant’s subjective SSQ score (averaged over all 17 responses) and objective RMS error (averaged across SNRs, environments, and sectors). An analysis of variance was also conducted to distinguish whether participant-chosen transducer type variation (circumoral and insert earphones) influenced the RMS error of participant responses. A type I error rate of 0.05 was used for all analyses, with *p* values considered when interpreting whether results were due to chance.

### 2.2. Results

**Figure 2:**
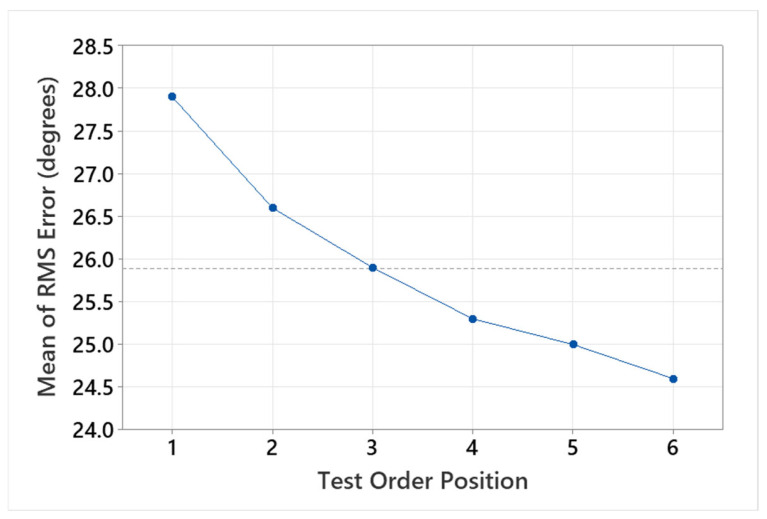
Mean participant response RMS error over position of condition (test) order showing mean improvement (3.3 degrees RMS error) despite inter-condition variables.

MAP testing was completed for all 30 participants under each of the six conditions (mean [range] duration 32 [26–39] min), with no difficulties under the remote testing conditions. Figure 2 shows the average RMS error of all participants across the test order positions. A large effect from the test order was observed, with participants showing a marked decrease in RMS error over time when averaged across test conditions. This equated to an average 3.3 degrees of improvement over the six test conditions. Correction factors corresponding to test order were applied (+0, +1.3, +2.0, +2.6, +2.9, and +3.3) prior to the further analysis of the test conditions.

**Figure 3:**
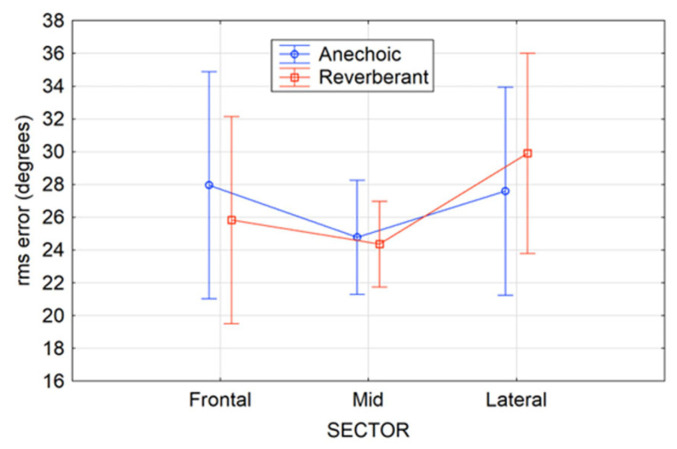
Mean RMS error (circles) within three sectors (frontal, middle, and lateral) in both an-echoic and reverberant conditions. The vertical bars denote 95% confidence intervals.

Table 1 shows results of a three-way ANOVA to examine the effects of the independent factors of the SNR, test environment, and presentation sector on RMS error. This showed no significant main effects (sector *p* = 0.14; environment *p* = 0.92; SNR *p* = 0.19), indicating that neither the SNR, environment, nor the sector significantly influenced localization accuracy. However, there was a statistically significant interaction between the sector and the environment F(2, 58) = 3.61, *p* = 0.031, indicating that the effect of the sector on RMS error depended on the simulated test environment. This is depicted in Figure 3, where participant RMS error was poorer in the anechoic condition compared to the reverberant condition when presentations originated from the frontal or mid sectors. However, within the lateral sector, average RMS error was poorer within the reverberant condition. All other two-way interactions were found to be statistically insignificant (sector × SNR *p* = 0.53; environment × SNR *p* = 0.82).

Figure 3 shows the average participant RMS errors for the three sectors (frontal, mid, and lateral) within each of the two environments. Participants demonstrated the lowest RMS error and the highest RMS error consistency within the middle sector in both anechoic and reverberant conditions.

**Figure 4:**
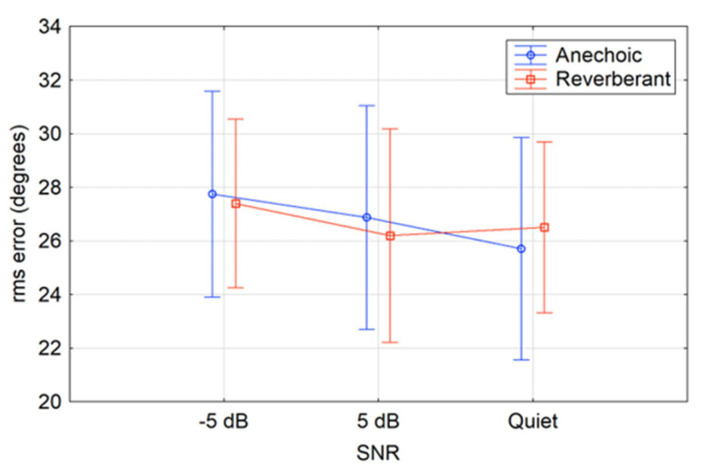
Mean RMS error (circles) within three individual SNRs (−5 dB, +5 dB, and quiet) in both anechoic and reverberant conditions. The vertical bars denote 96% confidence intervals.

Figure 4 shows the average participant RMS error for all three SNRs (−5 dB, +5 dB, and quiet) within each of the two environments. As expected, average performance was best in the anechoic and quiet condition; however, there was a minimal and non-significant effect from the simulated environment and SNR on average RMS error overall.

**Figure 5:**
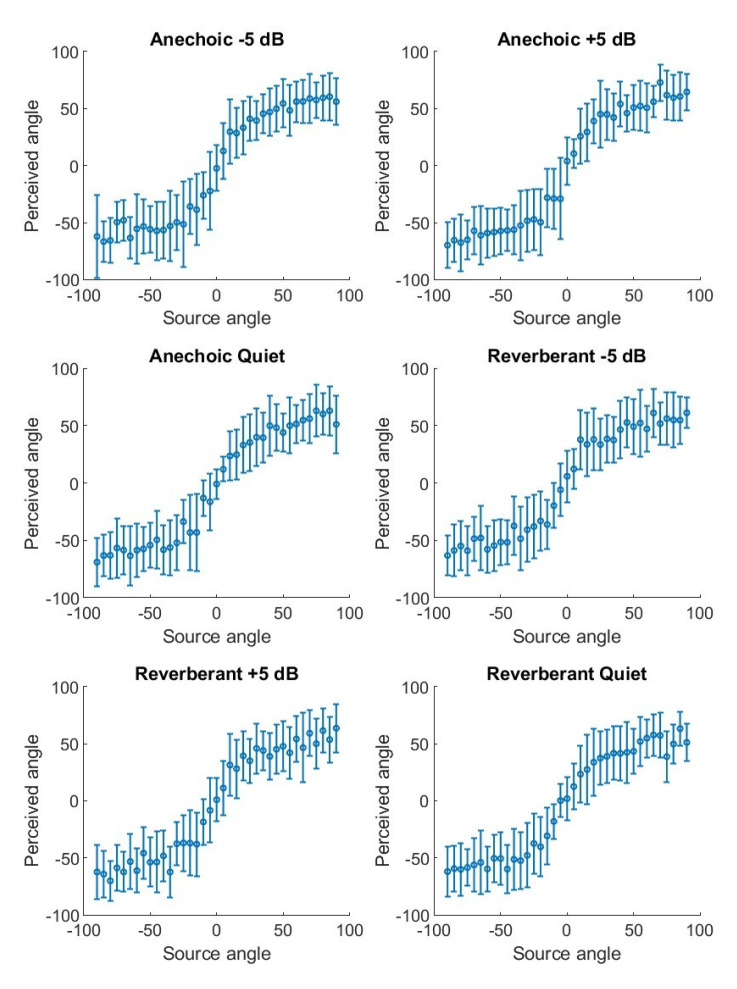
Perception function plots depicting perceived angle versus source angle. Circles show the mean, and vertical bars denote the standard deviation of perceived angles. Each graph represents responses in one of the six combinations of environment (anechoic or reverberant) and SNR (−5 dB, +5 dB, and quiet).

Given the non-significant influence of the sector, SNR, and environment on participant average RMS error, participant response RMS error values were averaged across all test conditions (following RMS correction for condition order). Overall, participants performed on the MAP test with moderate accuracy (mean RMS error = 25.91 degrees, SD = 6.43 degrees, SEM = 1.17 degrees, median = 25.29 degrees). Figure 5 depicts the mean participant responses across each test condition represented as a perception plot.

Table 2 shows the results of the second ANOVA to examine the effect of headphone type (circumaural vs. insert earphones) on participant RMS error (averaged across SNRs, environment, and sectors). This showed no significant impact of headphone type on average RMS error (headphone type *p* = 0.97), indicating that the type of headphone used for remote testing had no influence on participants’ ability to perform the localization task. Finally, there was no significant correlation between subjective responses on the spatial hearing sub-section of the SSQ and objective RMS error (r = −0.0015, *p* > 0.05).

**Figure 6:**
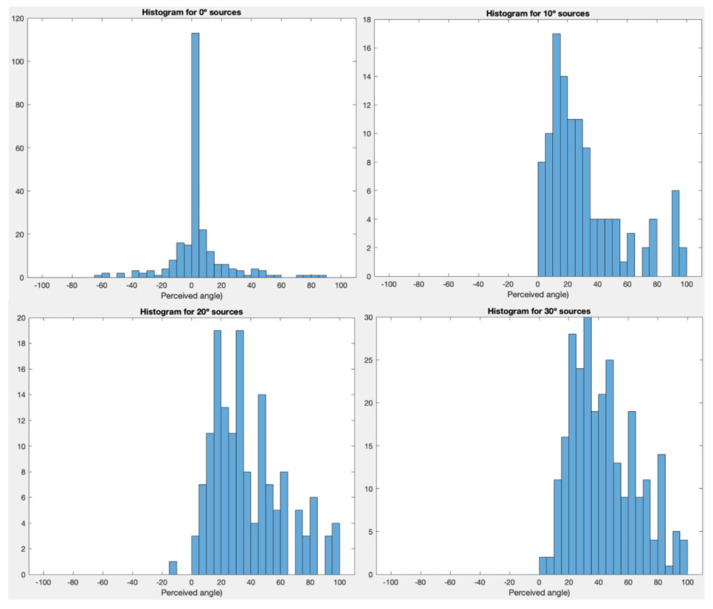
All participants perceived responses within every condition to various frontal source presentations (0, 10, 20, and 30 degrees, shown as graph headings) in the form of histograms. *Y*-axes vary in quantity corresponding to maximal participant response values. Appearance of front–back errors (responses at 95 and 100 degrees) are seen to appear once the source direction is almost directly in front.

The average RMS error for Experiment 1 was relatively high compared to free-field assessments (6–10 degrees) [4] of horizontal localization with similar designs. This inflation in RMS error may have occurred for several reasons, but was hypothesized to be due to front–back errors. Front–back errors are commonly seen in stationary horizontal localization tasks due to ITDs/IIDs arriving from the frontal hemisphere being the same as ITDs/IIDs arriving from the corresponding azimuth in the reverse hemisphere [33]. Figure 6 depicts all participant responses across each condition in relation to various source angles (0°, 10° 20°, and 30°). An increase in front–back error responses can be seen as the source angle diverged from the midline. In limiting the response array to the frontal hemisphere in Experiment 1, participants who perceived these presentations as being reversed likely chose the only available reverse hemisphere option, namely ±95 or 100 degrees, resulting in the inflation of the RMS error. This result prompted Experiment 2, which proposed that allowing participants access to a 360-degree response array would allow for greater correlation with the appropriate azimuth, albeit within the reverse hemisphere.

## 3. Experiment 2

### 3.1. Methods and Materials

#### 3.1.1. Participants

Twenty-eight young adults (71% female; mean [range] age 24 [21–29] years) were recruited from the University of Melbourne Audiology course’s 2021 and 2022 intakes. A sample size of a multiple of four allowed for a complete Latin Square design, with an equal number of participants tested under each combination of test conditions. Inclusion/exclusion criteria were the same as described for Experiment 1. Due to ongoing fluctuating COVID-19 restrictions, assessments were conducted face to face when possible (n = 18) and remotely when necessary (N = 10). Remote testing followed the Experiment 1 protocol. Written informed consent was obtained from all participants after approach via email.

#### 3.1.2. Conditions/Environment

The simulated environment techniques were the same as for Experiment 1 regarding both the listening environment and SNR. Due to the insignificant effect of the simulated environment or SNR on individual performance in Experiment 1, Experiment 2 also aimed to investigate whether more pronounced variations between test conditions would impact performance by increasing the reverberation time to 500 ms in the reverberant condition and varying the SNR between 0 dB SNR and 20 dB SNR.

Assessments again used the MAP protocol conducted under 4 different conditions, with the test order pre-determined using a Latin square design to control order effects. The four conditions comprised the following:

- Condition 1: Anechoic at 0 dB signal-noise-ratio;
- Condition 2: Anechoic at 20 dB signal-to-noise ratio;
- Condition 3: Reverberant at 0 dB signal-to-noise ratio;
- Condition 4: Reverberant at 20 dB signal-to-noise ratio.

#### 3.1.3. Most Comfortable Loudness Protocol (MCL) and Measurement of Azimuth Perception (MAP)

Prior to commencing the full MAP test protocol, participants were required to complete the MCL paradigm described in Experiment 1. Participants were again allocated a calibration factor, from which the signal intensity was calculated. Beyond this, an expanded test array and revised training protocol were introduced to address the issues highlighted in Experiment 1.

#### 3.1.4. Expanded Array

Experiment 2 used an expanded 360-degree virtual response array to allow for the participants to respond in both frontal and reverse hemispheres. Participants were presented with a similar test screen to that shown in Figure 1, but with a full 360-degree series of source angles occurring at 5-degree intervals, with 0 degrees corresponding to directly in front, 90 degrees directly to the right, −90 degrees directly to the left, and 180 degrees directly behind. The competing speech again came from five fixed locations, but these were now −144°, −72°, 0°, 72°, and 144°.

#### 3.1.5. Revised Training Protocol

Participants again completed a training protocol prior to assessment. The prior literature demonstrates a strong training effect with localization paradigms using non-individualized HRTFs, even when HRTFs differ significantly from the listeners’ own. As Experiment 1’s results show a likely learning effect with a systematic decrease in response RMS error when averaged across conditions, Experiment 2 used a revised training protocol to ensure participant familiarity with the test protocol and give the opportunity to practice translating the perceived source of azimuth from the simulated 3D environment into the corresponding source angle to support maximal performance under each condition. The protocol was given at the beginning of each assessment to ensure that no advantage was given to any one listening condition. Training was divided into two parts, with a demonstration followed by a practice sequence. The demonstration comprised a series of 20 presentations of the speech stimuli, presented under the designated test condition (environment and SNR), with corresponding source angles highlighted on screen with a face icon. Consecutive presentations of speech stimuli occurred at 20-degree intervals (0 degrees, 20 degrees, 40 degrees, etc.) for the full 360 degrees. Following the demonstration, the practice sequence commenced, with participants required to take control of the test screen and respond to a series of 12 speech stimuli, each originating from a randomized source angle. As in Experiment 1, prior to each presentation, “Listen Now” would flash on the screen to elicit attention. During practice, an icon highlighted the source angle, and participants self-selected the corresponding source angle.

#### 3.1.6. MAP Test

For the MAP assessment, participants were again given a remote control of the test screen and were presented with a series of 24 speech stimuli presentations under each condition. However, no visual aid was provided to guide participants to the source azimuth. The 360-degree array was coded to 8 different sectors, each with 9 possible source angles including the following: (1) Frontal-L, from 0 to −40 degrees, (2) Frontal Lateral-L, from −45 to −85 degrees, (3) Reverse Lateral-L, from −90 to −130 degrees, (4) Reverse-L, from −135 to −175 degrees, (5) Reverse-R, from 180 to 140 degrees, (6) Reverse Lateral-R, from 135 to 95 degrees, (7) Frontal Lateral-R, from 90 to 50 degrees, and (8) Frontal-R, from 45 to 5 degrees. Unknown to participants, the 24 presentations were programmed so that an equal number of presentations originated from within each sector, giving a total of 3 for each. As the training protocol for this experiment was longer, the number of presentations per condition was reduced to 24 to avoid participant fatigue and ensure the task could be completed within a practical timeframe. Prior to each presentation, “Listen Now” would flash on the screen to indicate when to focus. At the conclusion of each condition, participants were given a short break before completing the full MAP protocol again until all four conditions had been assessed. A measure of RMS error, reflecting the root mean square (RMS) value of the difference between the perceived source angle and the true source angle, was derived for each condition and automatically stored.

#### 3.1.7. Statistical Analysis

Prior to data analysis, RMS error scores for each condition were run through a front/back correction paradigm, a common practice in virtual localization tasks [33]. All source directions and perceived directions were reflected to the frontal hemisphere before calculating the discrepancy between the two directions. Minitab statistical software (version 19.2) [34] was used to determine the effects of the SNR and Simulated Test Environment (see Condition/Environment Simulation above) on the ability of participants to localize on the lateral plane, with measurements of RMS error being the dependent variable. A Two-way Repeated Measures Analysis of Variance (ANOVA) was conducted with the SNR and Test Environment as fixed factors and Subject as a random factor. Learning effects were investigated by plotting the mean performance of participants based on test order position regardless of the condition being examined.

### 3.2. Results

**Figure 7:**
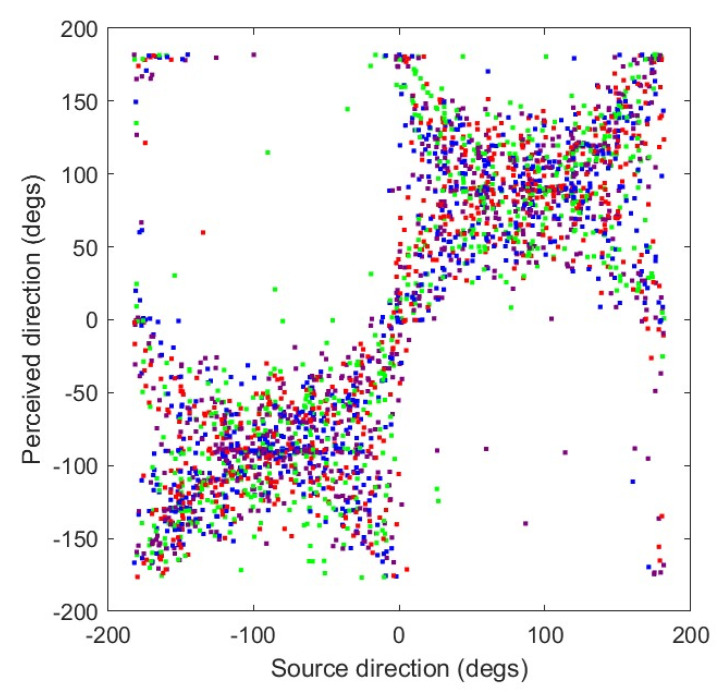
Perceived direction versus source direction for all conditions and participants. Color code: anechoic at 0 dB is red; anechoic at 20 dB is blue; reverb at 0 dB is green; reverb at 20 dB is purple.

MAP testing was completed for all participants with no difficulty. Figure 7 shows the perceived direction versus the source direction for all trials, participants, and conditions combined. The RMS localization error, averaged across all four conditions, was 59.7°. The largest localization errors in individual trials (i.e., far from the main diagonal) were the result of front–back confusions, which appear as the points clustered around the two lines at right angles to the main diagonal. Note that the small number of responses in the upper left and lower right corners are actually small localization errors, such as when the perceived direction is 180° for a source at −175°. Because of the marked effect of front–back reversals on RMS error, all source and response azimuths were reflected to the front hemisphere, and the RMS error was re-calculated. When averaged across all conditions for each participant, the RMS error was 27.2°, with a standard deviation of 5.0°. Errors based on frontally reflected azimuths were used for the remainder of the calculations.

**Figure 8:**
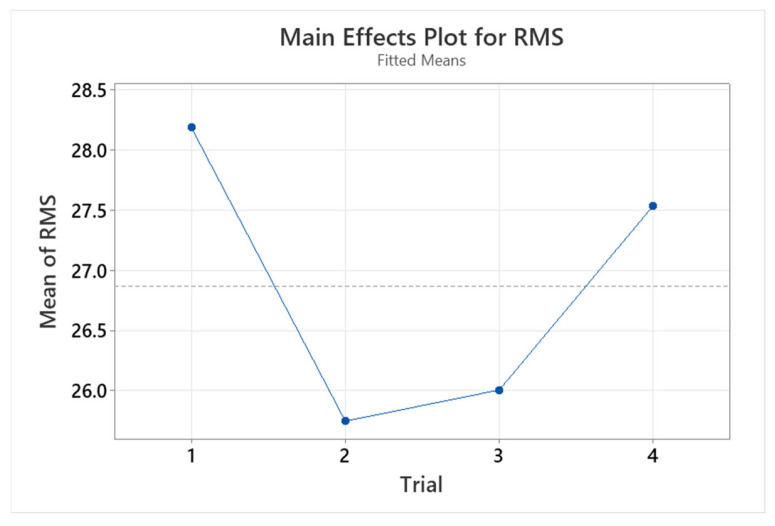
Mean participant response RMS error over position in the test order.

Figure 8 shows the average RMS error over positions in the test order for all participants. The results in this figure suggest that participants performed best in the second and third conditions. However, the average improvement was <3°, an insignificant difference. There were no significant learning effects throughout the test paradigm, with no marked improvement in performance in sequential test conditions when this was averaged across conditions.

**Figure 9:**
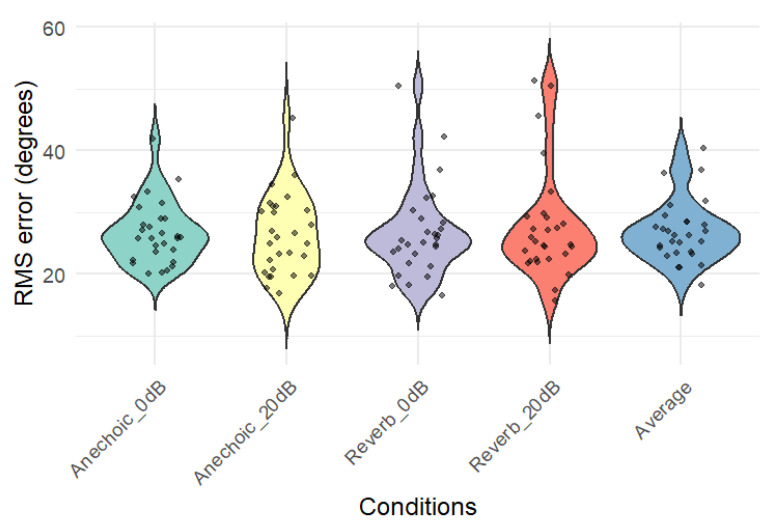
Distribution of RMS errors for each of the four conditions and for the RMS errors averaged across the four conditions.

Figure 9 shows the distribution of RMS errors for each condition and for the scores averaged across all conditions. The scores are approximately normally distributed, with the exception of those of one to three participants in each condition whose scores were markedly worse than the remaining participants. There was significant overlap between the individuals with poor performances in each condition.

**Figure 10:**
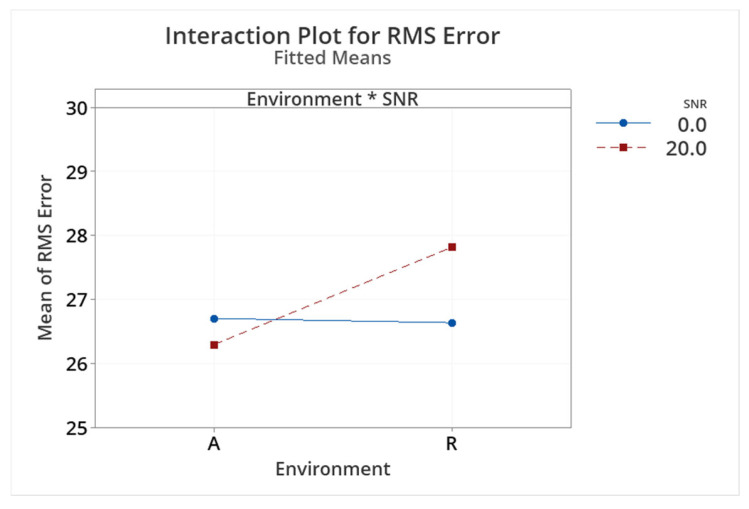
Mean participant response RMS error for two factors of test environment (A, Anechoic; R, Reverberant) and SNR (0 dB and +20 dB).

Figure 10 shows the average RMS error for all participants under both SNRs (0 dB and +20 dB) in both test environments (anechoic and reverberant). At an SNR of 0 dB, the test environment had a minimal effect on participants’ abilities to perform the task well. Although Figure 10 suggests that, at an SNR of +20 dB, participants appeared to perform better under anechoic conditions, the difference was only 1.5° and was not significant. Table 3 shows the results of a two-way repeated measures ANOVA investigating the effects of the SNR and test environment. Variations in the SNR and test environment did not influence participants’ abilities to localize in the horizontal plane under the headphone-based task (SNR *p* = 0.73; test environment *p* = 0.51). Additionally, there was no significant interaction between the SNR and the test environment (*p* = 0.47).

Table 4 shows the mean, standard deviation, and range of scores for each condition and for the scores averaged across all four conditions.

The significant F-value in the ANOVA for *Subject* indicates that, averaged across conditions, there were significant localization differences in the abilities of different participants. Consistent with this, RMS errors for three of the conditions were moderately and significantly correlated with each other, as shown in Table 5.

## 4. Discussion

The overall aim of the present study was to develop and evaluate the efficacy of a novel assessment of absolute localization under headphones for individuals with normal hearing, with the intention of implementing this assessment in a future APD test battery. There were three primary findings. A longer training protocol was found to be advantageous in eliminating the learning effect associated with non-individualized HRTFs. Secondly, reverberation and background masking noise had no significant effects on participant response RMS error, and, finally, RMS error was noted to be significantly inflated compared to previously reported free-field tasks [4,35].

### 4.1. Training

In Experiment 1, there were significant learning effects, with participant performance improving with consecutive tests (when averaged across environment and SNR). The lack of a significant practice effect in Experiment 2 is beneficial for use of the test clinically, where neither time nor attention span are available for the people being tested to gradually reach their final ability. The small average improvement seen real-time in Experiment 1 could be due to participant adaptation to the use of non-individualized HRTFs or to their increased ability to relate the directions heard to the graphical representation of the direction on the computer screen. Either of these explanations are consistent with the results of Hartmann [36], who found no short-term training effect on localization ability when tested under true free-field conditions. It therefore seems likely that the longer multi-stage training protocol administered in Experiment 2 resulted in the absence of a training effect.

Training, or adaptation capability, under localization protocols utilizing non-individualized HRTFs has been consistently observed in the literature [37,38,39]. The rate and quality of adaptation is influenced by the length of exposure to the stimuli and the incorporation of feedback, with stronger adaptation observed when visual feedback is incorporated. When two streams of sensory information are available, the brain will rely on the stronger of the sensory systems [40]. By using a visual stimulus alongside the training protocol, despite the conflicting nature of the auditory and visual stimuli, the visual system takes precedence. This phenomenon, often referred to as the ventriloquism effect, is strongest when two signals are temporally aligned and often has an after-effect [3]. When using non-individualized HRTFs, despite binaural cues that may vary from the listeners’ own neural templates, the temporally aligned visual reinforcement will assist with faster adaptation to the available binaural cues. This is supported by the absence of a learning effect in Experiment 2, which suggests that the visually reinforced training protocol may have successfully mediated maximal, or at least consistent, performance across test conditions. Other potential strategies for overcoming the short-term adaptation phase would be to use individualized HRTFs, or non-individualized HRTFs that are similar to that of the listeners’ own, which are known to produce better results in simulated auditory environments. However, the process of recording an individual’s HRTF and implementing them into a virtual auditory assessment would be costly and likely infeasible for a clinical test [41].

### 4.2. SNR

The results of this study also show that variations to SNR, limited to a range from 0 to +20 dB SNR, had no significant influence on participants’ ability to localize on the horizontal plane. A minimal impact of noise has been shown in other studies [25,42,43]. However, notable deterioration in localization ability has been observed when SNR ranges from 0 to +5 dB, and localization becomes noticeably poorer below −5 dB [44,45]. Any severe impact of SNR on localization has been shown to be more apparent in the plane of elevation [46]. These studies have also observed that the effect of SNR can be highly dependent on (1) the location of the masker and signal in the 3D environment, (2) the frequency components of masker and signal, and (3) the type of signal used for both the stimuli and masker [42,47,48], with a more pronounced effect of masking for continuous broadband stimuli (as opposed to speech-based stimuli), which may be due to speech stimuli containing more robust IID and ILD cues. Our pilot testing in paradigm development resulted in a decision to utilize −5 dB SNR in experiment 1, as we noted anything more adverse than this began to impact detection rather than localization ability. Although Lorenzi, Gatehouse, and Lever [42] suggested that localization degradation shows a monotonic relationship with SNR once negative ratios are used, and that any impacts of detection are only seen at close to −9 dB SNR and below (interpreted by performance consistent with chance levels), their study utilized steady white noise as a masker. It is hypothesized that the nonsignificant effect of noise on average RMS error performance at even −5 dB SNR levels in the current study was due to the listener’s ability to utilize the temporal characteristics of binaural glimpsing between the rapid fluctuations in a frequency that occurs in speech babble masking [49]. However, despite the ability of individuals with normal hearing to maintain accurate absolute azimuthal localization in poor SNRs, decreased performance in absolute localization tasks in individuals with hearing loss as SNR decreases has been reported [42,44,45]. It is important to note that results under the current paradigm may be influenced by the random error generated through translating the auditory signal into a response on a computer screen in real-time, unlike free-field tasks that typically require translation of the auditory signal into the physical space. For this reason, it is possible that poorer SNRs, compared to free-field tasks, may be required before an impact is observed on localization ability.

### 4.3. Environment

Both experiments showed no impact from simulated reverberation on participants’ ability to perform the task. When localizing, individuals rely on ILD and IID cues. In reverberant conditions, as the time course of a signal progresses, the added reflections of a signal result in a decorrelation of the ILD between the ears [50]. The non-significant effect of reverberation was thought to be primarily due to the nature of reverberation time length RT60; however, even for moderate reverberation times (RT60 = from 0.5 to 1 s), localization remains unaffected [36,44,51], as the auditory midbrain is able to efficiently encode the arriving direct sound’s onset (precedence effect) for spatial cues [50]. As a result, the direct signal arriving at the ear, which remains faithful to true binaural timing cues, can be used to extract the true sound source angle [50]. The strength of early reflections, as well as ongoing diffuse reverberation, on the other hand, does impact localization [52]. Both cohorts in the current study have demonstrated that individuals with normal hearing can perform consistently in both moderate- and high-reverberation environments, with no significant perceptual impacts on azimuthal localization capability. Longer reverberation times (RT60 = >1.0) have been shown to impact localization ability under similar tasks [44]; however, as previously noted, the inflation of RMS error associated with the random error generated from translating perceived angle to a computer screen may indicate that greater reverberation times may be required to observe a notable effect from the environment under a novel paradigm. Importantly, the lack of effect from reverberation for normal-hearing adults does not necessarily imply that reverberation time will have no effect for children with an auditory processing disorder, or even for typically developing children. Given the significant correlations between scores on three out of the four conditions, but not including the Anechoic−0 dB condition, this condition might not be the one that provides the most representative estimate of a person’s general localization ability.

### 4.4. Sector and SSQ

The significant finding of an interaction between the sector and environments’ *p* values (0.031) is likely spurious given that there is no physiological, neurological, or psychological basis for absolute localization performance to improve in reverberant and anechoic environments in the frontal and lateral azimuths.

### 4.5. Inflation of Scores Compared to Free-Field Tasks

The current study observed a higher average RMS error compared to similar tasks of absolute horizontal localization tested in the free field (Van den Bogaert et al. [53] mean RMS = 6.8°; Yost et al. [54] mean RMS = 5.98°). The fact that RMS error is higher under the current paradigm compared to what has been reported in some free-field studies may have multiple explanations. Under a headphone-based task, participants are prevented from accessing dynamic cues of auditory localization [55]. The occurrence of front–back errors within localization tasks, virtual or free-field, is commonly seen [33], and was observed across both experimental cohorts. The real-world resolution of front–back errors is primarily achieved through accompanying head movement, as different angular changes occur with such movement between hemispheres [56] and is almost always accompanied by the eyes [57]. Given the lack of access to dynamic cues and visual feedback in the current paradigm, it is therefore reasonable to anticipate the presence of front–back errors. However, the presence of these errors does not impact the viability of the assessment, which has been shown to be sensitive enough to detect individual variations in localization capacity within the current normal hearing adult cohorts. Therefore, the detection of localization deficits in the pediatric and/or APD positive populations is promising.

As previously noted, it is hypothesized that, in Experiment 1, when perceiving a reversed presentation, participants opted to report the only available reverse hemisphere option available (+/−95 or 100 degrees). Experiment 2 gave access to the full response array, allowing participants to accurately report their perceived angle, and permitting front–back correction calculations (reassigning front–back errors back to the correct hemisphere) within the test paradigm. With front–back errors corrected, the average RMS error was essentially equivalent between the two experiments. However, the opportunity to analyze both corrected and uncorrected scores highlights the value of using an expanded test array, regardless of the outcome.

### 4.6. Limitations of This Study

Beyond the influence of front–back errors, it is likely that the lack of visual reinforcement, unfamiliarity in interpreting virtual signals, and use of non-individualized HRTF may have contributed to a higher RMS error than is typically obtained in true free-field conditions. Localization of a sound source, while heavily reliant on binaural auditory cues, also receives significant contributions from other sensory systems [58]. In real-world conditions, sound source localization is almost always complemented by movements of the eyes and head [57], which reinforces the information received through the auditory pathways. In a true free-field localization task, front–back errors can be simply resolved with a head turn, as it easily becomes apparent whether the source is then to the left or the right. More generally, participants may be more easily able to interpret auditory signals as they are being represented within the observer’s physical space. However, in the current test paradigm, this ability is entirely absent. In future, it will be important to consider that head movement has the potential to occur when testing children. Adaptive angular change manipulation HRIRs is possible under visually enforced paradigms (VR) [59]; however, this would be costly to implement. It is possible that the current results represent the best achievable performance under virtual conditions suitable for clinical implementation with technology that is likely to be available in clinics for some time.

While technology and inter-connectivity are improving at an exponential rate, internet-based perception research is relatively new. Despite the potential and drawbacks discussed here, the practice is well set out by Woods et al. [60] and is not only possible, but may be useful for research purposes as well as in clinical environments. The ultimate goal for future research would be in transporting the listener into a realistic 3D sound environment under headphones without any disruption by conflicting, and therefore disconnecting, head-movement relation to angular change and in relation to visual cues.

## 5. Conclusions

The results of this study demonstrate the ability of normal-hearing individuals to perform at a consistent level of acuity during a novel virtual localization task, supporting the possibility of translating virtual auditory simulation technology into the clinical environment. Our results suggest that the MAP protocol may be a viable option as a test of absolute localization to be included in an APD test battery. The test has sufficient sensitivity to detect individual differences in ability, even among young adults with no known listening difficulties.

Future research in this area should investigate the performance of children with normal hearing under the MAP protocol to better understand the viability of such an assessment on this demographic and to further investigate the performance of those reporting a localization deficit. Directional audiometry has been requested since Nordlund [61]. Our paradigm meets calls for this to be better achieved in the virtual space [25]. Appropriate correction for noise/reverberation parameters, front–back errors, and learning effects should continue to be explored, with encouraging potential for future research paradigms and clinical assessment related to APD localization deficit.

## Figures and Tables

**Table 1:** F ratio table for the three-way analysis of variance (ANOVA) on the effects of SNR, test environment, and presentation sector on RMS error.

Effect	SS	DF.	MS	F	*p*
**Sector** Error	1577.0 22,128.8	2 58	788.5 381.5	2.07	0.14
	
**Environment** Error	0.8 2177.0	1 29	0.8 75.1	0.0104	0.92
	
**SNR** Error	203.5 3485.9	2 58	101.7 60.1	1.69	0.19
	
**Sector × Enviro** Error	448.4 3533.0	2 58	224.2 60.9	3.68	0.031
	
**Sector × SNR** Error	134.7 7347.7	4 116	33.7 63.3	0.532	0.71
	
**Enviro × SNR** Error	53.9 1897.4	2 58	27.0 32.7	0.824	0.44
	
**Sector × Enviro × SNR** Error	302.1 4864.1	4 116	75.5 41.9	1.80	0.13
	

**Table 2:** F ratio table for the analysis of variance (ANOVA) on the effect of headphone type (circumaural and insert earphones) on RMS error.

Effect	SS	DF	MS	F	*p*
Headphones Error	0.05 819.08	1 28	0.0.5 29.25	0.0016	0.97
	

**Table 3:** Summary of results from the two-way repeated measures ANOVA investigating the effect of the SNR and test environment on individuals’ RMS errors.

Source	DF	Adj SS	Adj MS	F	*p*
Subject	27	2690.32	99.642	2.98	0.00
Environment	1	14.79	14.792	0.44	0.51
SNR	1	4.16	4.163	0.12	0.73
Enviro × SNR	1	17.60	17.596	0.53	0.47
Error	81	2707.37	33.424		
Total	111	5434.24			

**Table 4:** Descriptive statistics for scores for each condition and for scores averaged across all four conditions, all with front–back errors corrected.

	Anechoic		Reverberant		Average
	*0 dB SNR*	*20 dB SNR*	*0 dB SNR*	*20 dB SNR*	
Mean	26.7	26.3	26.6	27.8	26.9
SD	5.0	6.5	7.3	8.9	5.0
Minimum	20.1	16.8	16.6	15.6	18.3
Maximum	41.9	45.4	50.5	51.3	40.4

**Table 5:** Correlations between the RMS errors for the four conditions. Each cell shows the correlation coefficient, with the corresponding *p*-value in parentheses. Values with *p* < 0.05 are shown in bold.

	Anechoic 0 dB	Anechoic 20 dB	Reverb 0 dB	Reverb 20 dB
**Anechoic 0 dB**		0.10 (0.62)	0.14 (0.49)	0.01 (0.96)
**Anechoic 20 dB**	0.10 (0.62)		**0.62 (<0.001)**	**0.41 (0.033)**
**Reverb 0 dB**	0.14 (0.49)	**0.62 (<0.001)**		**0.58 (0.001)**
**Reverb 20 dB**	0.01 (0.96)	**0.41 (0.033)**	**0.58 (0.001)**	

**Figure A1:**
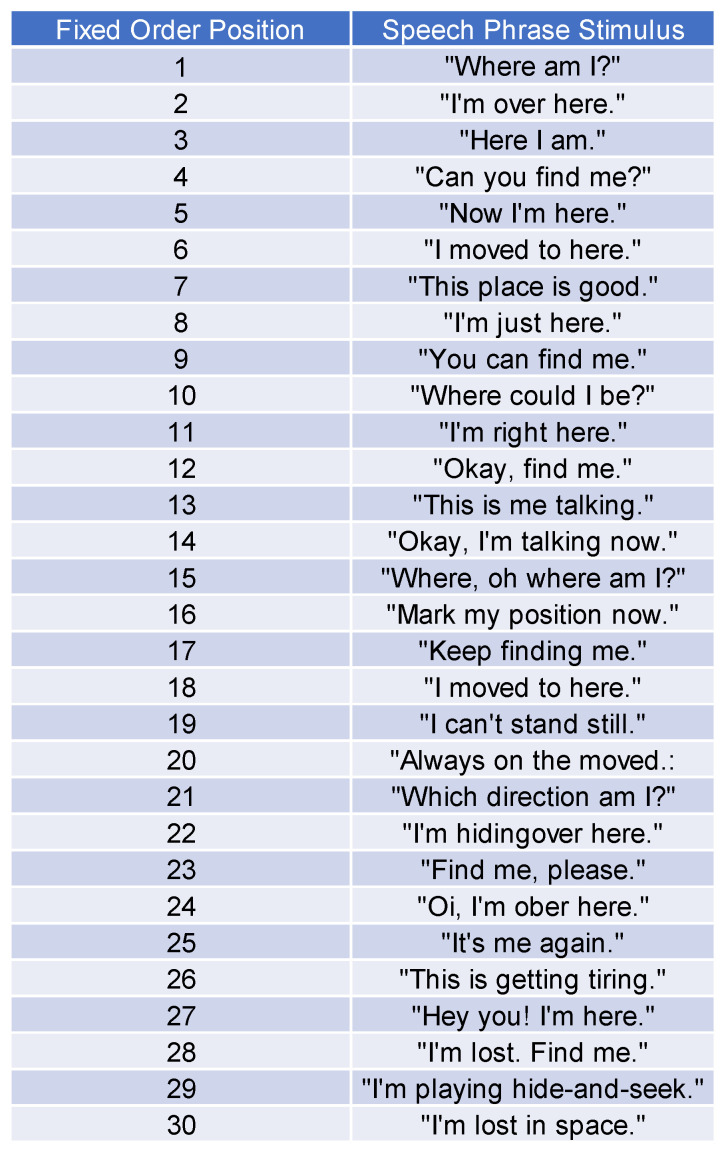
Speech phrases used as source stimuli in Measurement of Azimuthal Perception (MAP) test. Speech phrases were presented in a fixed order.

## Data Availability

The datasets presented in this article are not readily available due to technical limitations. Requests to access the datasets should be directed to Jack Hargreaves.
